# Dietary restrictions in endurance runners to mitigate exercise-induced gastrointestinal symptoms

**DOI:** 10.1186/s12970-020-00361-w

**Published:** 2020-06-10

**Authors:** Jill A. Parnell, Kim Wagner-Jones, Robyn F. Madden, Kelly Anne Erdman

**Affiliations:** 1 Department of Health and Physical Education, Mount Royal University, 4825 Mount Royal Gate SW, Calgary, Alberta T3E 6K6 Canada; 2 Helios Wellness Centres, Teaching, Research, Wellness Building, Suite 402, 3280 Hospital Drive NW, Calgary, AB T2N 4Z6 Canada; 3 Faculty of Kinesiology, 2500 University Drive NW, University of Calgary, Calgary, Alberta T2N 1N4 Canada; 4 University of Calgary, Sports Medicine Centre, 2500 University Drive NW, Calgary, Alberta T2N 1N4 Canada

**Keywords:** Running, Food intolerances, Exercise-induced gastrointestinal symptoms, Runner’s gut, Gastrointestinal tolerance, Pre-exercise meal, Dietary restrictions

## Abstract

**Background:**

Endurance runners frequently experience exercise-induced gastrointestinal (GI) symptoms, negatively impacting their performance. Food choices pre-exercise have a significant impact on the gut’s tolerance to running, yet little information is available as to which foods runners restrict prior to exercise.

**Methods:**

A questionnaire designed to assess dietary restrictions pre-racing and gastrointestinal symptoms was administered to 388 runners. Fisher’s exact tests determined differences in gender, age, performance level, and distance with follow-up multivariable logistic regression modeling.

**Results:**

Runners regularly avoided meat (32%), milk products (31%), fish/seafood (28%), poultry (24%), and high-fiber foods (23%). Caffeinated beverages were commonly avoided in events 10 km or less (*p* < .001); whereas in females, increased running distance was a predictor of avoiding high-fiber foods (OR = 6.7; 95% CI = 1.6–28.5). Rates of food avoidance were elevated in younger and more competitive runners. Common GI symptoms included stomach pain/cramps (42%), intestinal pain/discomfort (23%), side ache/stitch (22%), urge to defecate (22%), and bloating (20%). The prevalence of GI symptoms was higher in younger athletes, especially females, which may explain their propensity to avoid foods. Lower recreational athletes were the least likely to report GI symptoms. Diarrhea incidence increased with running distance. **Conclusions:** Identification of voluntary food restrictions in the pre-running meal highlights trends that can direct further research.

## Background

Gastrointestinal (GI) symptoms are a common cause of underperformance in athletes, yet are less frequently considered in nutrition recommendations, which focus on fluid, macronutrient, and micronutrient intakes. The prevalence of exercise-induced GI disturbances varies depending on methodology and ranges from 30 to 90% amongst endurance runners [1]. Commonly reported GI symptoms include flatulence, bloating, diarrhea, urge to defecate, belching, reflux/heartburn, abdominal pain/cramping, nausea, vomiting, fecal blood loss, and bloody diarrhea [1–5].

Underlying factors promoting GI symptoms during physical activity are multifaceted and include physiological, nutritional, mechanical, and psychological factors [1, 4, 6–9]. Physiological causes are attributed to two pathways: 1) circulatory-gastrointestinal, which involves a reduction of splanchnic blood flow during exercise, and 2) neuroendocrine-gastrointestinal pathway where there is an increase in sympathetic activation, thus reducing GI function [8]. Splanchnic hypoperfusion can result in intestinal ischaemia, which can increase intestinal permeability, heightening bacterial translocation and promoting inflammation [6, 8]. High intensity exercise may also decrease gastric motility and emptying [1, 10–12]. This cascade of physiological events can increase the potential for nutrient malabsorption [9] further aggravated by extreme environmental conditions [13, 14]. Exercise-induced nutrient malabsorption could result in increased small intestinal water content and gas production due to bacterial fermentation, as well as activation of the “ileal break” feedback mechanism [8].

Nutritional factors surround food selection of an athlete prior to exercise and have the potential to reduce or exacerbate exercise-induced GI symptoms yet remain largely unstudied. Given the limited data exploring endurance athletes’ food and fluid intolerances pre-training and competition, nutrition professionals are challenged to make recommendations. Broadly, endurance runners are advised to determine their own pre-exercise food intolerances with general advice to avoid foods high in fat, protein, and fiber, as well as limit concentrated sources of carbohydrates [15–17]. Anecdotally, many endurance athletes believe a specific food and/or fluid prior to exercise can increase GI symptoms. For example, 41% of non-celiac athletes followed a gluten-free diet more than 50% of the time, partly to reduce GI symptoms [18]. Evidence for the benefit of a low fermentable oligosaccharides, disaccharides, monosaccharides and polyols (FODMAP) diet to reduce exercise-induced GI symptoms is emerging [19–21]; however, there remains a paucity of research in pre-exercise nutrition. Dehydration is believed to aggravate symptoms [9], whereas others suggest gut-training via carbohydrate ingestion during running may be beneficial [22].

Mechanical factors include the motion of the sport [1, 8]. Finally, psychological factors should be considered: as an association between exercise-related GI distress and stress and anxiety has been reported [7]. Considering that individuals will most easily be able to control nutritional factors, the purpose of this study was to assess voluntary, pre-exercise food restrictions related to running-induced GI symptoms and differences related to gender, age, performance level, and event.

## Methods

### Participants

The questionnaire was administered to endurance runners 18 years of age or older. Athletes were recruited from running groups, races, and at pre-race events across southern Alberta. A required sample size of 377 was calculated for the survey, based on a margin of error of no more than 5% with a 95% confidence level [23]. Participants who reported food allergies or celiac disease were excluded, as were those with other GI disorders such as inflammatory bowel disease (IBD), irritable bowel syndrome (IBS), heartburn/reflux, gallbladder removal, etc. to limit confounding variables. These individuals will be assessed separately in future publications. Those who reported a specific food intolerance were removed from the analysis of that particular food to ensure that the food avoidance was related to exercise and not a general aversion; however, were included in the remainder of the analyses. The study received ethical approval from the Mount Royal University Human Research Ethics Board (ethics ID 2016–38). All participants provided written, informed consent.

### Questionnaire

The researchers approached participants either at a running clinic, event package pick-up or after completing a running event and asked them to complete a paper version of the questionnaire. The questionnaire was developed to collect information on basic demographics, running experience, medical conditions, voluntary food restrictions, exercise-induced GI symptoms experienced if they consume a trigger food, reasons for avoiding foods, and sources of information. The questionnaire asked participants to select options for each question by checking boxes. There was also the option for an open-ended “other”. The questionnaire was validated by experts in the field and tested for reliability in a test/re-test manner with 39 participants [24]. A copy of the questionnaire is available in supplemental file 1.

### Statistical analysis

Athlete responses were categorized into groups based on gender, age (young athletes 18–34 years; masters athletes 35 years and older), performance level (lower recreational defined as “don’t compete” or “lower half of age group”; upper recreational defined as “upper half of age group”; and competitive athletes defined as “provincial, national or international”) and most frequent race distance (“don’t compete”; “5 km”; “6 to 10km”; “11 to ½ marathon [21km]”; “> ½ marathon [> 21 km]”). Given that participants were primarily recruited from race events, those selecting “don’t complete” were grouped with those who selected lower recreational, as it was assumed that for these individuals while they were competing in the event, they didn’t consider themselves competitive. We also recruited a small number of participants from running clinics, in which case participants may not have been competing in events despite training for running. Information is presented as the number and percentage of total athletes. Significant differences between groups (gender, age, race distance, performance level) in the percent of athletes who consumed or avoided pre-exercise foods, symptoms experienced, and reasons for avoiding foods were determined by a Fisher’s exact test with *p* < .05 considered statistically significant. Multivariable logistic regression modeling was conducted to examine the relationship between more than one demographic/performance characteristic (gender, age, race distance, performance level) and each outcome variable. Similar to the bivariate analysis, the outcomes included: food avoidance variables (only those variables where over 10% of athletes reported avoiding a specific food) and all GI symptom variables. The possibility of interaction between each pair of demographic/performance variables was considered first before including any variable individually, and the significance of an interaction term was assessed using the Likelihood Ratio Statistic. Interaction variables were considered significant and included if *p* < .05. If there was no significant interaction, demographic/performance variables were still eligible for inclusion in the model if they were significant at *p* < .10 in an individual logistic regression model with the food avoidance/GI symptom outcome variable. Variables were retained in the multivariable model if *p* < .05. Odds ratios (OR) and 95% confidence intervals (CI) are reported for significant findings. A *p*-value <.05 was considered to be statistically significant. Given that multiple comparisons were made with a 5% level of significance, there is a risk of false positives. Data were analyzed using SPSS statistical software version 25 (IBM, Armonk, New York, USA) or STATA S/E Version 15 (StataCorp LLC, College Station, TX, USA).

## Results

Five hundred and thirty runners completed the questionnaire; however, 142 were removed due to reported food allergies or GI associated medical reasons. Analysis was based on 388 participants [44% male; mean age 41 years (SD 13)]. Response rates ranged from 89 to 100% of the questions. If a participant chose not to answer a question (e.g. gender), their answers to the remaining questions were included in the analyses. Masters athletes comprised 63% of the sample. Regarding performance level, 35% self-classified as lower recreational, 56% as upper recreational, and 9% as competitive. For run distances, 4% don’t compete, 17% 5 km, 33% 6-10 km, 35% 11–21 km, and 11% 21+ km. Participants reported diabetes (*n* = 3), asthma (*n* = 2), high blood pressure (*n* = 2), autistic spectrum disorder (*n* = 1), autoimmune disorder (*n* = 1), high cholesterol (*n* = 1), hyperthyroidism (*n* = 1), and migraines (*n* = 1).

### Foods avoided - gender and age

**Fig. 1:**
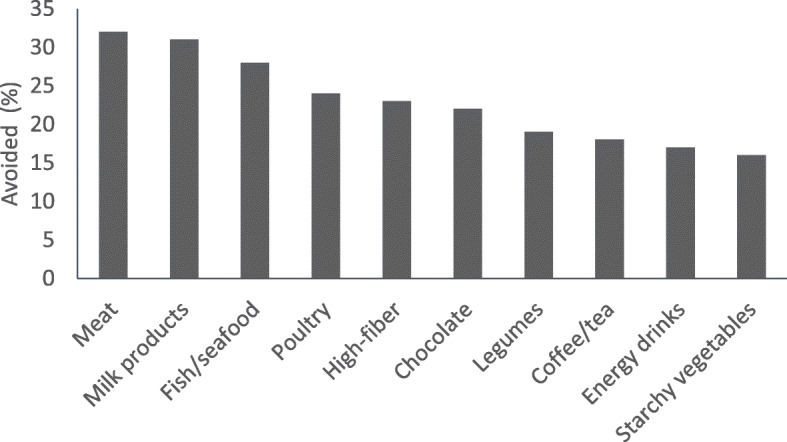
Pre-race food avoidances in runners to minimize exercise-induced GI symptoms. Percentage of endurance runners who avoid a food category pre-race to minimize GI symptoms

The most commonly avoided foods pre-racing included meat, milk products, fish/seafood, poultry, foods high in fiber, chocolate, legumes, coffee/tea, energy drinks, and starchy vegetables (Fig. 1). Differences between gender/athlete age categories are presented in Table 1.

**Table 1:** Frequently avoided foods pre-race by gender and athlete age group

Foods Avoided	Young Males n(%)	Masters Males n(%)	Young Females n(%)	Masters Females n(%)	*p*-value
Meat	21 (42)	28 (26)	22 (28)	40 (34)	.186
**Milk products**	**17 (35)**	**22 (22)**	**26 (45)**	**25 (26)**	**.017**
Fish/seafood	18 (36)	27 (26)	24 (30)	31 (27)	.557
Poultry	15 (30)	22 (21)	17 (21)	31 (26)	.519
High-fiber	13 (25)	24 (22)	15 (18)	32 (26)	.529
**Chocolate**	**17 (33)**	**13 (12)**	**23 (27)**	**29 (24)**	**.009**
**Legumes**	**14 (27)**	**14 (13)**	**10 (12)**	**29 (25)**	**.025**
Coffee or tea	7 (14)	13 (12)	21 (25)	23 (20)	.092
**Energy drinks**	**15 (30)**	**10 (9)**	**16 (20)**	**16 (14)**	**.009**
Starchy vegetable	10 (19)	15 (14)	15 (18)	16 (13)	.633
**Lactose-free milk**	**14 (27)**	**10 (9)**	**12 (15)**	**14 (12)**	**.029**
Eggs	12 (23)	10 (9)	12 (15)	14 (12)	.117
**Soy milk**	**13 (25)**	**7 (7)**	**10 (13)**	**16 (13)**	**.016**
Sports drink	7 (14)	11 (10)	3 (4)	16 (14)	.085
Vegetables	8 (15)	7 (7)	7 (9)	15 (13)	.284
Cold cereal	5 (10)	9 (8)	9 (11)	15 (13)	.749
Coconut milk	12 (23)	9 (8)	7 (8)	12 (10)	.050
**Almond milk**	**12 (23)**	**7 (7)**	**8 (10)**	**11 (9)**	**.025**

Foods commonly avoided pre-racing by gender and classification as young or masters athlete. Individuals with a self-declared intolerance to a food category were removed from the analysis for that food. Percentages represent the frequency of individuals from each category who report avoiding the food. Differences between groups were determined by a Fisher’s exact test. Significant differences *p* < .05 are bolded

Significant differences were found in milk products and energy drinks, which were more commonly avoided in younger athletes. Masters males had the lowest avoidance of chocolate; whereas, young males were the most likely to avoid the milk alternatives and lactose-free milk (Table 1). Additional multivariable analysis found gender and running distance to be an interaction in their relationship with avoidance of high-fiber foods such that females running longer distances (anything greater than 5 km) were more likely to avoid high-fiber foods pre-racing than males running longer distances (OR = 6.7; 95% CI = 1.6–28.5).

### Foods avoided - event and performance level

The most commonly avoided foods by event in the pre-race period are presented in Table 2.

**Table 2:** Frequently avoided foods pre-race by race distance

Foods Avoided	5 km n(%)	6–10 km n(%)	11–21 km n(%)	22+ km n(%)	*p*-value
Meat	18 (28)	37 (31)	40 (31)	18 (43)	.404
Milk products	15 (27)	41 (39)	28 (25)	9 (25)	.110
Fish/seafood	18 (27)	35 (29)	32 (25)	16 (39)	.416
Poultry	14 (21)	32 (26)	27 (21)	14 (33)	.350
**High-fiber**	**14 (21)**	**22 (18)**	**34 (26)**	**17 (41)**	**.024**
Chocolate	12 (18)	31 (25)	30 (23)	8 (19)	.757
Legumes	10 (15)	20 (16)	26 (20)	13 (31)	.176
**Coffee or tea**	**19 (29)**	**31 (25)**	**15 (12)**	**1 (2)**	**<.001**
Energy drinks	15 (23)	21 (17)	16 (13)	6 (14)	.269
Starchy vegetable	12 (19)	20 (16)	18 (14)	6 (14)	.864
Lactose-free milk	11 (17)	18 (15)	13 (10)	6 (14)	.502
Eggs	10 (15)	17 (14)	14 (11)	6 (15)	.744
Soy milk	9 (14)	16 (13)	14 (11)	6 (14)	.879
Sports drink	9 (14)	16 (13)	11 (9)	2 (5)	.360
Vegetables	4 (7)	12 (10)	13 (10)	9 (22)	.111
Cold cereal	13 (20)	11 (9)	13 (10)	4 (10)	.172
Coconut milk	9 (14)	13 (10)	11 (9)	7 (17)	.379
Almond milk	9 (14)	12 (10)	10 (8)	6 (14)	.430

Foods commonly avoided pre-racing by race distance. Individuals with a self-declared intolerance to a food category were removed from the analysis for that food. Individuals, who selected “don’t compete” as their race distance were removed from the analyses. Percentages represent the frequency of individuals from each category who report avoiding the food. Differences between groups were determined by a Fisher’s exact test. Significant differences *p* < .05 are bolded

Pre-racing, high-fiber foods were more commonly avoided in the marathon and ultra-marathon distances, whereas, coffee/tea was avoided more often in races that were 10 km or shorter. Smoothies were more often avoided by those competing in the longest races 5 km (11%), 6-10 km (7%), 11 km–21 km (5%), > 21 km (26%); *p* = .001. Multivariable regression modeling did not detect any interactions except for high-fiber, as noted previously.

Frequently avoided food by performance level pre-racing are presented in Table 3.

**Table 3:** Frequently avoided foods pre-race by performance level

Foods Avoided	Lower Recreational n(%)	Upper Recreational n(%)	Competitive n(%)	*p*-value
Meat	30 (24)	71 (34)	14 (41)	.061
Milk products	26 (25)	56 (31)	14 (47)	.085
Fish/seafood	**23 (18)**	**67 (33)**	**13 (38)**	**.007**
Poultry	22 (18)	59 (28)	8 (24)	.082
High-fiber	25 (19)	51 (24)	12 (35)	.148
Chocolate	22 (17)	57 (27)	5 (15)	.068
Legumes	18 (14)	41 (19)	10 (30)	.101
**Coffee or tea**	**25 (20)**	**42 (20)**	**1 (3)**	**.031**
Energy drinks	15 (12)	37 (18)	8 (24)	.181
Starchy vegetable	19 (15)	33 (16)	5 (15)	1.000
Lactose-free milk	11 (9)	34 (16)	7 (21)	.077
Eggs	11 (9)	33 (16)	5 (15)	.154
Soy milk	13 (10)	30 (14)	5 (15)	.564
Sports drink	10 (8)	29 (14)	3 (9)	.270
**Vegetables**	**6 (5)**	**27 (13)**	**7 (21)**	**.007**
**Cold cereal**	**7 (6)**	**31 (15)**	**3 (9)**	**.021**
Coconut milk	9 (7)	27 (13)	6 (18)	.123
Almond milk	8 (6)	27 (13)	5 (15)	.120

Foods commonly avoided pre-racing by performance level. Individuals with a self-declared intolerance to a food category were removed from the analysis for that food. Lower recreational includes those who selected “don’t compete” and “lower recreational”. Percentages represent the frequency of individuals from each category who report avoiding the food. Differences between groups were determined by a Fisher’s exact test. Significant differences *p* < .05 are bolded

In general, lower recreational athletes were less likely to avoid foods, with significant differences showing for fish/seafood, vegetables, and cold cereal. Conversely, competitive athletes were less likely to avoid coffee or tea (Table 3). Athletes who classified themselves as lower recreational were also less likely to avoid bars/gels (3% lower recreational, 13% upper recreational, 9% competitive; *p* = .013), nuts (6% lower recreational, 11% upper recreational, 21% competitive; *p* = .046) and juice (6% lower recreational, 14% upper recreational, 6% competitive; *p* = .027). Statistical modeling results showed that, after adjusting for confounding by age and gender, higher performance level was a significant predictor of avoidance of meat (upper recreational OR = 1.8; 95% CI = 1.1–3.0 and competitive OR = 2.5; 95% CI = 1.1–6.0). Performance level was also an independent predictor with athletes racing in upper recreational level more likely to avoid chocolate (OR = 2.2; 95% CI = 1.2–3.9). Competitive athletes were significantly more likely to avoid lactose-free milk compared to lower recreational athletes (OR = 2.3; 95% CI = 1.1–4.7). Upper recreational/competitive athletes were more likely to report avoidance of eggs pre-racing (OR = 2.3; 95% CI = 1.1–4.9). Competitive athletes were more likely to avoid almond milk (OR = 1.1; 2.3; 95% CI = 1.1–5.4).

### GI symptoms - gender and age

**Fig. 2:**
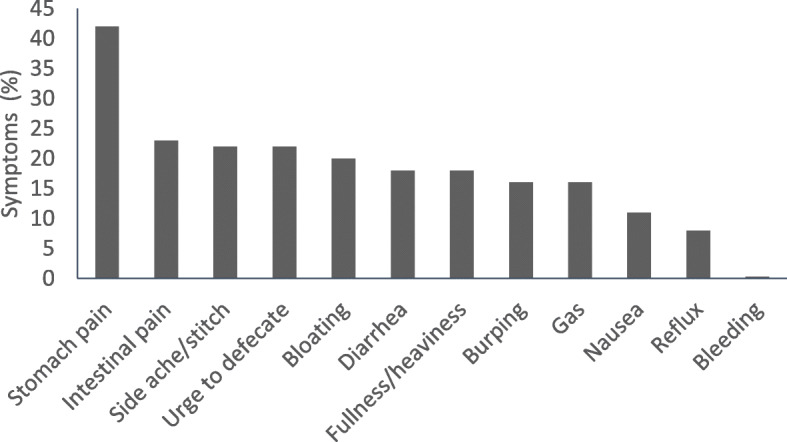
Symptoms experienced while racing. Symptoms that runners reported they would experience during a race if they consumed a food that they would typically avoid. Data is presented as percentage of all runners

Runners primarily reported: stomach pain/cramping, intestinal pain/discomfort, side ache/stitch, urge to defecate, bloating, diarrhea, and fullness/heaviness if they consumed an aggravating food pre-race (Fig. 2). Difference between gender/age groups are presented in Table 4.

**Table 4:** Symptoms experienced pre-racing by gender and athlete age group

Symptoms	Young Males n(%)	Masters Males n(%)	Young Females n(%)	Masters Females n(%)	*p*-value
**Stomach pain/cramps**	**25 (48)**	**35 (33)**	**47 (57)**	**50 (41)**	**.009**
Intestinal pain/discomfort	15 (29)	18 (17)	20 (24)	34 (28)	.171
**Side ache/stitch**	**18 (35)**	**14 (13)**	**28 (34)**	**20 (17)**	**<.001**
Urge to defecate	9 (17)	16 (15)	21 (25)	35 (29)	.055
**Bloating**	**13 (25)**	**10 (9)**	**25 (30)**	**23 (19)**	**.002**
Diarrhea	5 (10)	16 (15)	18 (22)	28 (23)	.114
**Fullness/heaviness**	**9 (18)**	**12 (12)**	**24 (31)**	**17 (16)**	**.015**
**Burping/belching**	**13 (25)**	**8 (8)**	**22 (27)**	**15 (12)**	**.001**
**Gas**	**5 (10)**	**18 (17)**	**22 (27)**	**13 (11)**	**.016**
**Nausea/vomiting**	**7 (14)**	**7 (7)**	**17 (21)**	**10 (8)**	**.017**
Reflux/heartburn	6 (12)	7 (7)	8 (10)	8 (7)	.580
Bleeding	0 (0)	0 (0)	1 (1)	0 (0)	.372

Exercise-induced gastrointestinal symptoms experienced by runners while racing if they consume an offending food. Percentages represent the frequency of individuals from each category who report they would experience the symptom if they ate a food they would typically avoid. Differences between groups were determined by a Fisher’s exact test. Significant differences *p* < .05 are bolded

Runners also reported urge to urinate and phlegm in the open ended “other” category. Pre-racing, young females suffered from exercise-induced GI symptoms more frequently than other groups. They experienced the highest levels of stomach pain/cramps, bloating, heaviness/fullness, gas, and nausea/vomiting (Table 4). After adjusting for performance level in logistic regression analysis, females, irrespective of age, and athletes competing at upper recreational/competitive levels were significantly more likely to report the urge to defecate (females OR = 1.9; 95% CI = 1.2–3.1) (upper recreational / competitive OR = 2.1; 95% CI = 1.2–3.7). After adjusting for running distance, females and athletes running the longest distances were significantly more likely to report diarrhea (females OR = 2.6; 95% CI = 1.5–4.4; greater than half marathon OR = 4.7; 95% CI = 1.9–11.3).

### GI symptoms - performance level and event

Pre-racing upper recreational athletes had the highest rates of stomach pain/cramping at 49% (*p* = .018). Additionally, the higher the performance level, the more frequently the urge to defecate during runs was reported (competitive athletes 41%; *p* = .014). After examining gender, age, and running distance in multivariable logistic regression modeling with performance level, it is of note that upper recreational and competitive athletes compared to lower recreational athletes were significantly more likely to report side ache/stitch (OR = 2.0; 95% CI = 1.1–3.6). Diarrhea incidence increased with distance run (*p* = .008). No other symptoms during racing were associated with running distance after adjusting for gender, age, and performance level in the analysis.

### Reasons for food-avoidances

**Fig. 3:**
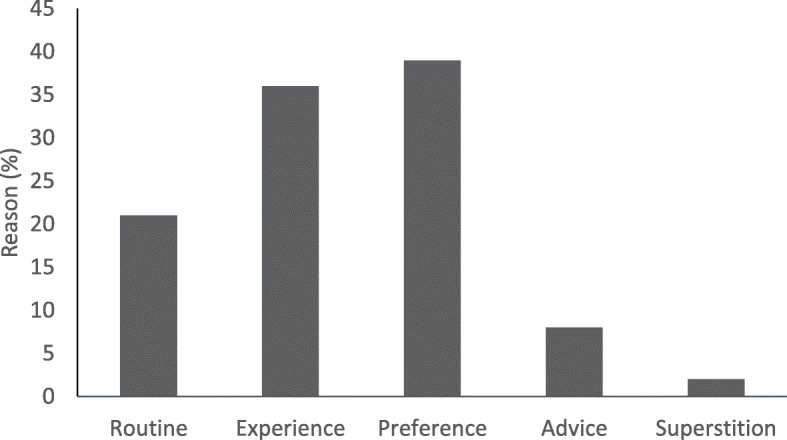
Reasons for pre-race food avoidances in runners. Reasons endurance runners choose to avoid a food pre-race. Data is presented as percentage of all runners

Reasons for avoiding foods are provided in Fig. 3. The most common reasons listed for food avoidances included personal experience and personal preference. Advice from others, including health professionals, was indicated as a reason for a food avoidance 8% of the time.

## Discussion

Our research provides insight into voluntary pre-exercise food restrictions endurance runners use to mitigate GI symptoms, via a fully powered, reliable and validity tested questionnaire. Furthermore, we controlled for GI disorders, allergies, and food intolerances. Finally, we have representation from a diverse group of runners, allowing for increased specificity in our understanding of food avoidances.

### Pre-exercise food restrictions

Foods most commonly avoided were milk products, high protein, high-fiber, chocolate, and caffeinated beverages, aligning with recommendations to limit protein, fat, and fiber [15–17] while concurrently highlighting the need for more in-depth research. Importantly, each food item is a complex mix of macro and micronutrients as well as other bioactive ingredients that can impact GI symptoms; thus, caution is advised when making conclusions regarding an individual nutrient in a food. Generally, the higher the performance level, the more likely an athlete was to restrict food, which is likely a function of increased GI symptoms with increasing exercise intensity [8, 25]. Another possibility is that higher level athletes are more experienced and therefore more familiar with aggravating foods.

Avoidance of milk and milk products was common among runners in our study. Interestingly, others have found that in female runners declaring they have an “outstanding diet”, intake of dairy beverages was significantly lower than those who rated their diet as “average” [26]. Dairy products are complex foods and naturally contain lactose. We are in agreement with others who have demonstrated that athletes will remove sources of lactose; a high FODMAP food, from their diet to improve GI symptoms [27]. Not all dairy products contain lactose, therefore, future research should subdivide this category into lactose containing and lactose-free products. Considering that lactose-free milk was also among the top foods avoided, the reasoning is likely multifaceted. Dairy products are also sources of fat and protein, which are thought to promote exercise-induced GI symptoms [16]. Further analysis indicated that young females were most likely to avoid milk products followed by young males, which is in agreement with other findings indicating age as a negative predictor for dairy consumption, in the general population [28]. Our research suggests that age remains a predictor for dairy avoidance in sport, even when allergies and intolerances are considered. Further, Yantcheva et al. [28] report the perception of mucous production as a common reason for the avoidance of dairy, which may be related to reports of phlegm or mucus as an “other” symptom. The role of dairy in mucus production has not been fully elucidated [29]; however, it clearly remains a popular perception.

Foods typically considered high protein, especially animal protein, were commonly avoided pre-exercise. Athletes are advised to avoid excessive protein pre-exercise [16]; however, recommendations for the appropriate amount of protein and studies regarding the effects of protein intake on exercise-induced GI symptoms in runners are lacking. Tiller et al. [15] recommend 1.6 g/kg body weight per day as a minimum for ultra-endurance runners; however, they do not specifically address the pre-race period. They do note that protein intake during specifically ultra-endurance running may positively affect energy metabolism and mitigate muscle damage with the caveat that the results are equivocal. Snipe et al. [30] explored 14.8 g whey protein intake pre/during a 2 h run, designed to induce exertional heat stress, as compared to water or 15 g glucose. A reduction in intestinal epithelial injury and intestinal permeability was found with both whey protein and glucose; however, gut discomfort and gastrointestinal symptoms were higher with protein. In basketball players, the addition of protein to the pre-exercise meal at 1 g/kg body weight resulted in increased gastrointestinal symptoms as compared to carbohydrate alone [31]. The aforementioned studies support our conclusions regarding protein rich foods and increased exercise-induced GI symptoms. Given the potential performance benefits of protein in the pre-exercise meal and popularity of high-protein diets, additional research is required to determine if there is a threshold below which symptoms are minimal. Generally, the higher the performance level, the more likely athletes were to avoid high protein foods, possibly due to increased GI symptoms with increased exercise intensity [8, 25].

Runners avoided high-fiber foods, which aligns with others describing lower intakes of dietary fiber by endurance runners, although not specifically in the pre-exercise meal [32, 33]. Limiting of dietary fiber pre-exercise is advised [16, 17], based largely on a study by Rehrer et al. [34], linking dietary fibers to intestinal cramps. Dietary fiber ingestion is associated with decreased splanchnic vascular resistance resulting in increased splanchnic vasodilation and splanchnic flow. These physiological effects oppose blood flow needs during exercise, where there is prolonged splanchnic hypoperfusion, and consequently may present as abdominal disturbance [35]. Delayed gastric emptying with viscous dietary fibers [36] may also play a role, further exacerbated by high intensity exercise [37]. Conversely, insoluble fibers stimulate peristalsis via fecal bulking [36], which may promote urge to defecate or diarrhea. Fiber restriction was more common in longer distances, which may reflect changes in GI transit time, as diarrhea was reported more frequently in longer distances. Finally, many fiber rich foods are high FODMAP foods, which may provide another mechanistic explanation. Dietary fibers are heterogeneous and vary in their physiological effects; thus, recommendations should consider fiber type in addition to quantity. Further, given the health benefits of adequate fiber and recent advances in the understanding of the importance of the gut microbiota in athletes [6], research is required regarding pre-exercise timing strategies to optimize intakes while minimizing GI symptoms.

Dark chocolate has been proposed as an ergogenic aid via increased nitric oxide [38]; however, caution should be advised given our results of high avoidance pre-race, although we did not distinguish between the different types of chocolate. Chocolate has been described as a food item that provokes GI disturbance, particularly constipation [39], although research in athletes is lacking. Chocolate contains several biologically active compounds including cocoa, caffeine, and fat; thus, the mechanisms are unclear. It is known, however, that high fat foods may aggravate exercise-induced GI symptoms [16].

Coffee and tea represent another food group often avoided and morning caffeine intake has been associated with increased GI symptoms in the lower gut of triathletes [40]. Interestingly, competitive athletes and longer distance runners were less likely to avoid coffee/tea and this may be a reflection of the potential ergogenic effects of caffeine in endurance exercise [41]. Future research should consider the effects of coffee, tea, and herbal infusions separately as they contain different constituents and can result in different physiological effects.

Energy beverages are a cocktail of vitamins, sugars, and plant extracts, especially stimulants. GI upset is included in the list of commonly reported symptoms after energy drink use [42] and safety is a concern [43]. Energy drinks were restricted more often in younger athletes; however, they are a relatively new product and marketing of energy drinks is typically youth oriented. It is possible that older athletes would not report avoiding energy drinks if they were unfamiliar with the product.

### Exercise-induced gastrointestinal symptoms

GI disturbance during runs is a common concern as described here and throughout the literature [1, 7–9]. At a minimum, GI symptoms associated with exercise are related to mechanical forces, altered GI blood flow, changes in the GI mucosal activity, neuroendocrine changes, and stress [1, 4, 7–9].

Female runners were more likely to experience urge to defecate and diarrhea. Additionally, young females reported highest rates of gas, nausea, fullness, and stomach pain/cramps, which supports research examining GI symptoms in an exclusively female running cohort where younger age was related to increased GI symptoms [44]. Further, others support a higher prevalence of GI symptoms in female athletes [3, 22, 45, 46]. Conversely, in a trial to determine the effect of biological sex on GI symptoms during exertional-heat stress, by timing testing during the follicular phase of the menstrual cycle, no differences in GI symptoms were reported except for flatulence and abdominal stitch, which were higher in males [47]. The aforementioned findings suggest further research is required to determine the causes of increased symptoms in females and the potential relationship to sex hormones and female gut physiology.

The higher prevalence of symptoms in younger athletes is confirmed by the literature [44–46]. Increased age may protect against GI symptoms due to diminished splanchnic vasoconstriction via reduced catecholamine response and consequently increased oxygen supply [44]. Further, increased age often reflects increased running experience, which is associated with fewer GI symptoms [44, 45].

Considering performance level, it was often the lower recreational athletes who were least likely to report symptoms. Potentially these athletes are competing at lower intensities, thus have fewer symptoms, as GI symptoms are reported to increase with exercise intensity [8, 25]. Symptoms are thought increase with distance [2], however, studies are required, and are likely compromised by the tendency to consume food and fluid during the longer events. In our study, diarrhea increased with the longest distances run after controlling for other factors.

When the reasons for food avoidances were explored, the majority of the participants relied on personal experience or personal preference. Further investigation into their sources of information and how this varies by age, gender, event, and performance level is of interest.

### Limitations

A limitation to the study is its observational nature, which precludes any causal conclusions. Conversely, the study does highlight candidate foods for future clinical trials, as it is not feasible to test every food in a controlled study. The potential confound of a food intolerance was considered by removing those individuals with reported food intolerances; however, this was not always clear for combination foods such as smoothies or high-fiber foods. Fortunately, food intolerances were typically clearly identified. With respect to food categories: fats, oils, spicy foods, and high FODMAP foods should be added to future questionnaires. An “other” section, where people reported avoiding high fat and spicy foods, was included, suggesting these are areas of concern. Additionally, we did not ask participants to indicate the severity of their symptoms or provide a symptom for each food avoidance; thus, we cannot associate a specific food to a specific symptom or comment on the degree of discomfort. Finally, multiple comparisons were made with a 5% level of significance; thus, there is a risk of false positives.

## Conclusions

A complete understanding of strategies endurance runners use in their pre-exercise meal, to minimize GI symptoms associated with runner’s gut, is essential for improving athletes’ performance and comfort. The identification of food avoidance trends will direct future clinical trials designed to identify specific foods endurance runners can consume to minimize GI symptoms and optimize performance.

## Supplementary information

**Additional file 1.**

## Data Availability

The datasets used and/or analysed during the current study are available from the corresponding author on reasonable request. The questionnaire is available as a supplemental file (see Additional file 1).

## Abbreviations

CI: Confidence interval
FODMAP: Fermentable oligosaccharides, disaccharides, monosaccharides and polyols
GI: Gastrointestinal
IBD: Inflammatory bowel disease
IBS: Irritable bowel syndrome
OR: Odds ratio
